# Effects of different bicarbonate on *spirulina* in CO_2_ absorption and microalgae conversion hybrid system

**DOI:** 10.3389/fbioe.2022.1119111

**Published:** 2023-01-06

**Authors:** Pengyu Zhang, Qian Sun, Ye Dong, Shaohan Lian

**Affiliations:** ^1^ Tianjin Building Materials Science Research Academy Co. Ltd, Tianjin, China; ^2^ Tianjin Key Laboratory of Indoor Air Environmental Quality Control, School of Environmental Science and Engineering, Tianjin University, Tianjin, China

**Keywords:** Spirulina platensis, CO_2_ absorption, NaHCO_3_, KHCO_3_, CO_2_ absorption hybrid with microalgae conversion

## Abstract

According to the characteristics of power plant flue gas emission and the requirements of reducing CO_2_ capture cost. CO_2_ absorption hybrid with microalgae conversion (CAMC) can avoid the challenges of heat consumption during absorbent desorption and nutrient consumption during microalgae culture. In this study, the bicarbonate solution (represents the products of CO_2_ absorption by Na_2_CO_3_ and K_2_CO_3_) is used as carbon source for mutagenic *Spirulina platensis* cultivation, and different concentrations of bicarbonate were set to explore the best carbon source. The results showed that NaHCO_3_ was a better medium for the CO_2_ absorption hybrid with microalgae conversion system, which was beneficial for the growth of mutagenic *Spirulina*, compared with K_2_CO_3_. When .3 mol/L NaHCO_3_ was added to the CO_2_ absorption hybrid with microalgae conversion system, the highest biomass dry weight, carbon fixation rate and carbon utilization efficiency were obtained, which were 2.24 g/L, 230.36 mg/L/d and 26.71%, respectively. In addition, .3 mol/L NaHCO_3_ was conducive to protein synthesis, reaching 1,625.68 mg/L. This study provided a feasible idea for power system to achieve carbon neutrality in the future.

## 1 Introduction

Nowadays, climate change caused by fossil fuel utilization has attracted more and more attention. As one of the largest carbon emission countries, China has claimed to achieve carbon peaking and carbon neutrality in 2030 and 2060, respectively. To realize these targets, CO_2_ capture, storage and utilization (CCUS) has been recognized as an important strategy (Ju et al., 2012; Kanno et al., 2017; Khoo et al., 2019). According to data from the International Energy Agency (IEA), the power sector accounts for nearly two-thirds of the increase in energy-related CO_2_ emissions, with more than 10 Gt CO_2_ from coal. CO_2_ capture and utilization in coal-fired power plants can effectively reduce carbon emissions in the power generation industry.

Chemical absorption, physical adsorption, membrane separation, low temperature distillation and microalgae bio-sequestration are commonly used carbon capture technologies (Song C. et al., 2019). Conventional CO_2_ utilization methods include chemical catalytic conversion to prepare chemicals, auxiliary production of petroleum, *etc.* Considering energy consumption and process integrity, the coupling of chemical absorption and biotransformation is a promising solution compared to other capture and utilization technologies (Song et al., 2019b). Bicarbonate is an intermediate product of conventional CO_2_ chemical absorption process and an important carrier of carbon source for microalgae. Therefore, a novel concept to combine CO_2_ absorption and microalgae culture, named as CO_2_ absorption and microalgae conversion (CAMC) system was proposed (Song et al., 2019b). The CAMC system with bicarbonate as the link can not only avoid energy consumption in the analytical process, but also solve the nutrient problem in the microalgae culture process, which is an economic and environmentally friendly carbon capture technology (Song et al., 2018). In CAMC system, the advantages of absorption and bioconversion could be combined and intensify CO_2_ capture and utilization efficiency.

The forms of inorganic carbon in medium include CO_2_, CO_3_^2-^, HCO_3_^−^, H_2_CO_3_, *etc.* However, it should be noted that not every kind of inorganic carbon can be utilized by microalgae, and the two forms of inorganic carbon that *Spirulina* can utilize are CO_2_ and HCO_3_^−^ (Pereira et al., 2019; Li et al., 2020). Compared with green algae, *Spirulina* has strong carbonic anhydrase (CA) activity and higher bicarbonate utilization efficiency (Chang et al., 2013; Cheng et al., 2017; de Jesus et al., 2018). Previous study used different kinds of absorbent to cultivate *Spirulina*, and investigated the growth of microalgae with three initial biomass concentrations in eight concentrations of monoethanolamine (MEA) and three concentrations of sodium hydroxide solutions (da Rosa et al., 2016). The results showed that the appropriate concentration of MEA did not inhibit the growth of *Spirulina*. Compared with sodium hydroxide, the concentration of inorganic carbon in MEA solution was doubled, and the protein content of *Spirulina* was 17% higher than that obtained by using sodium hydroxide. Therefore, different chemical absorbers could affect the performance of CAMC system. It is necessary to find economic and feasible chemical absorbers to improve the carbon sequestration efficiency of *Spirulina* in CAMC system.

Na_2_CO_3_, K_2_CO_3_ are widely used CO_2_ absorbers, due to their low toxicity, solvent loss and cost (Fang et al., 2020). Based on this, NaHCO_3_ and KHCO_3_, which could be generated after the full absorption of CO_2_ by Na_2_CO_3_ and K_2_CO_3_, were selected as the carbon sources for the cultivation of mutagenic *Spirulina*, and different carbon source concentrations (0.1 mol/L, .2 mol/L and .3 mol/L) were also set to get the best absorbent concentration. The effects of different absorbers on biomass and carbon sequestration efficiency of mutagenic *Spirulina* in CAMC system were studied. In addition, the potential value-added components such as protein, lipid and polysaccharide were also measured. CAMC system can provide guidance for the establishment of efficient biorefinery plant to produce high value-added products.

## 2 Materials and methods

### 2.1 Microalgae strain

Previous study has shown that *Spirulina* has a developed CO_2_ enrichment mechanism (CCM), which can actively pump enough HCO_3_^−^ into cells to improve intracellular CO_2_ concentration and promote carbon utilization efficiency (Li et al., 2020). In this study, the used microalgae were mutagenic *Spirulina platensis* which was from Zhejiang University.

### 2.2 Experimental procedure

Mutagenic *Spirulina platensis* was initially precultured with Zarrouk medium. After 5 days of culture, the mutagenesis *Spirulina platensis* in the logarithmic growth phase was used as inoculum for the formal experiment.

The chemical absorbents selected in this paper were Na_2_CO_3_ and K_2_CO_3_, respectively, and the corresponding NaHCO_3_ and KHCO_3_ were formed after fully absorbing CO_2_. .1 mol/L、0.2 mol/L and .3 mol/L NaHCO_3_ and KHCO_3_ were added to the basal medium as carbon sources, respectively, to investigate the effects of different carbon sources on the growth of mutagenic *Spirulina* in CAMC system, as shown in Table 1. Microalgae cultivation was preceded in 250 ml serum bottles with 200 ml culture medium. The incubator was placed at a constant temperature of 30°C ± 1°C and illuminated all day at a light intensity of 4,000 Lux. Three parallel experiments were performed in each group and shake the bottle at a set time each day. The biomass, pH, inorganic carbon and other experimental indexes were measured every 3 days.

**TABLE 1:** Batch cultivation condition in CAMC system.

Inorganic carbon	Concentration (mol/L)	Group
NaHCO3	.1	Na-.1 mol/L
NaHCO3	.2	Na-.2 mol/L
NaHCO3	.3	Na-.3 mol/L
KHCO3	.1	K-.1 mol/L
KHCO3	.2	K-.2 mol/L
KHCO3	.3	K-.3 mol/L

### 2.3 Analytical methods

The standard curve of mutagenic *Spirulina* biomass was obtained based on the linear relationship between dry weight and OD_560_. Firstly, dilute the algae solution with deionized water to OD_560_ = .2, .4, .6, .8, 1.0, and then measure 100 ml, respectively. The filter membrane with a pore size of .45 μm was dried in an oven at 105°C to a constant weight. Then the filter membrane was weighed and the weight was recorded. The measured 100 ml of algae solution was filtered through the membrane, and the membrane was again dried in an oven at 105°C to a constant weight and the weight was recorded. The difference between the two membrane weights was the dry weight of mutagenic *Spirulina platensis* biomass. The standard curve was as following:

$$X_{biomass}=0.404•{OD}_{560}-0.0016,R^{2}=0.9988$$ (1)

Carbon fixation rate (P_CO2_, mg/L/d) was calculated by referring to (Song et al., 2019c).

$$P_{CO2}\left(mg/L/d\right)=P_{biomass}\cdot C_{algae}\cdot \left(M_{CO2}/M_{C}\right)$$ (2)

Hereinto, P_biomass_ was the biomass yield (mg/L/d), C_algae_ was the carbon content in mutagenic *Spirulina*, M_CO2_ and M_C_ were the molar mass of CO_2_ molecule and C atom, respectively.

Carbon utilization efficiency was calculated according to the method mentioned by (Ding et al., 2017):

$$E_{C}=100\cdot C_{algae}\cdot X_{biomass}/\left[12/84\cdot D_{C}\right]$$ (3)

E_C_ represented carbon conversion efficiency (%), X_biomass_ represented the biomass dry weight (g/L), D_C_ represented the consumption of NaHCO_3_ or KHCO_3_ (g/L), and C_algae_ represented the carbon content of mutagenic *Spirulina platensis* (%).

The lipid content was determined by Nile red staining. 2.55 ml algae solution was added with 450 μL dimethyl sulfoxide and 24 μL Nile red solution. It was placed in a darkroom at 30°C for 10 min, and 580 nm was selected as the excitation fluorescence detection wavelength (Bertozzini et al., 2011).

20 mg of mutagenic *Spirulina* powder was added to the test tube, and 4 ml of deionized water was added to the water bath at 100°C for 2 h. The supernatant was then filtered through a vacuum pump, 1 ml of the filtrate was taken and 4 ml of ethanol was added, and it was placed in a refrigerator at 4°C for 12 h. Finally, the content of polysaccharide was determined by phenol sulfuric acid method (Gasljevic et al., 2008).

Chlorophyll a and b and carotenoids of mutagenic *Spirulina* were determined by spectrophotometer. The detail determination method is as follows: 5 ml uniform algal liquid was centrifuged at 5,000 rpm for 10 min. Then, the supernatant was removed, 5 ml methanol (90%) was added, extracted at 4°C for 24 h, then centrifuged at 5,000 rpm for 10 min, the supernatant was taken, and the absorbance of the supernatant was measured at 665, 652 nm and 470 nm by UV–visible spectrophotometer. The contents of chlorophyll a and b and carotenoids in microalgae were calculated by using the Formulas (Eqs. 4–6).

$$Chlorophyll\;a\;\left(mg/L\right)=16.82\;A_{665}\;–\;9.28\;A_{652}$$ (4)

$$Chlorophyll\;b\;\left(mg/L\right)=36.92\;A_{652}\;–\;16.54\;A_{665}$$ (5)

$$C_{carotenoid}\;\left(mg/L\right)=\left(1000\;A_{470}\;–\;1.91\;C_{a}\;–\;95.15\;C_{b}\right)/225$$ (6)

After the cultivation and harvesting, the algae powder was dried, and the algae powder was analyzed and measured. The proportion of N element in biomass N_algae_ (%) was obtained. The content of protein X_protein_ (mg/L) was calculated according to the relationship between nitrogen content and protein production multiplied by the coefficient 6.25. X_biomass_ (g/L) was the dry weight of mutagenic *Spirulina platensis* biomass at the end of the cycle (Li et al., 2020).

$$X_{protein}=X_{biomass}\cdot N_{algae}\cdot 6.25$$ (7)

One-way analysis of variance (ANOVA) was used for statistical analysis. Experimental results are expressed as mean ± standard error. The mean is based on parallel experiments and is within a 95% confidence interval.

## 3 Results and discussion

### 3.1 Dry weight variation of mutagenic *spirulina* biomass

**FIGURE 1:**
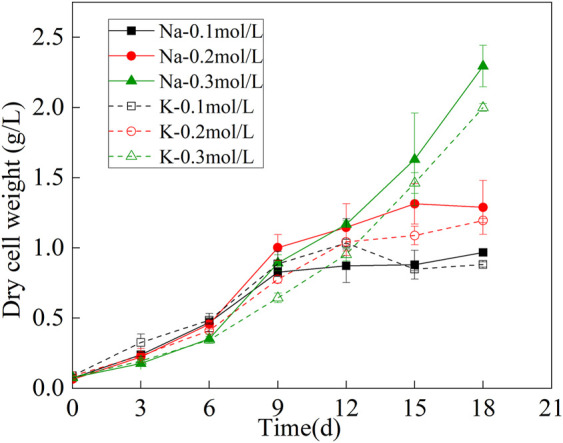
Dry weight variation of microalgae biomass under different bicarbonate concentrations.

The biomass dry weight curve was shown in Figure 1. The growth of mutagenic *Spirulina* in the CAMC system with different inorganic carbon concentrations were similar in the first 9 days, indicating that the three concentration ranges of NaHCO_3_ and KHCO_3_ (.1, .2, .3 mol/L) could maintain the growth of mutagenic *Spirulina* in the CAMC system. After the 9th day, the growth trend of mutagenic *Spirulina* began to show obvious changes. The biomass of mutagenic *Spirulina* with .1 mol/L and .2 mol/L NaHCO_3_ and KHCO_3_ tended to be stable, while the biomass of mutagenic *Spirulina* with .3 mol/L NaHCO_3_ and KHCO_3_ still increased rapidly. After 18 days of culture, the biomass dry weight of Na-.3 mol/L group was the highest, reaching 2.29 g/L, while that of K-.3 mol/L group was 2.00 g/L. Therefore, compared with K_2_CO_3_, Na_2_CO_3_ as a CO_2_ chemical absorber was more conducive to the growth of mutagenic *Spirulina* in CAMC system, and the optimal concentration was .3 mol/L.

### 3.2 Change of pH in CO_2_ absorption hybrid with microalgae conversion system

**FIGURE 2:**
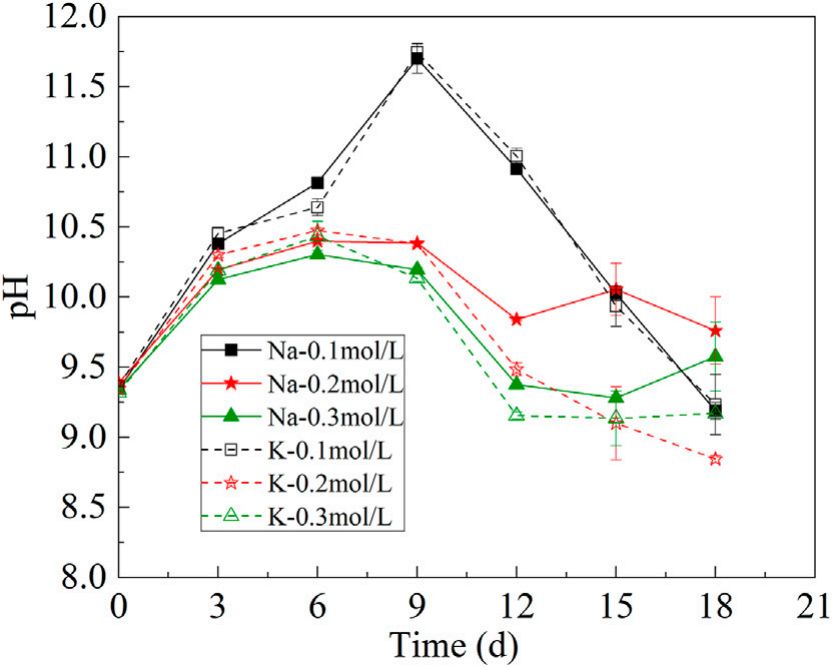
Change of pH under different bicarbonate concentrations.

The change of pH in the CAMC was shown in Figure 2. HCO_3_^−^ will enter cells by active transport under the action of transporters, and the pH of the solution will change with the consumption of HCO_3_^−^. After HCO_3_^−^ enters the cell, it will react with intracellular H^+^, leading to an increase in the concentration of OH^−^ in the internal environment of cell. H^+^ in extracellular solution will transmembrane neutralize OH^−^ in cells, leading to an increase in pH (Chi et al., 2013). This process involves one or more CA that facilitate the conversion between HCO_3_^−^ and CO_2_. Therefore, the pH of each group showed an upward trend during the first 9 days of culture. However, the pH of the CAMC system began to decrease on 9^th^ day. This could be caused by more CO_3_^2-^ in the system, which absorbed CO_2_ from the air. After 18 days of culture, the pH of different groups was stable between 8.5 and 10, but the range of pH change was relatively stable in the CAMC system with .3 mol/L HCO_3_^−^.

### 3.3 Changes of chlorophyll a content in mutagenic *spirulina*

**FIGURE 3:**
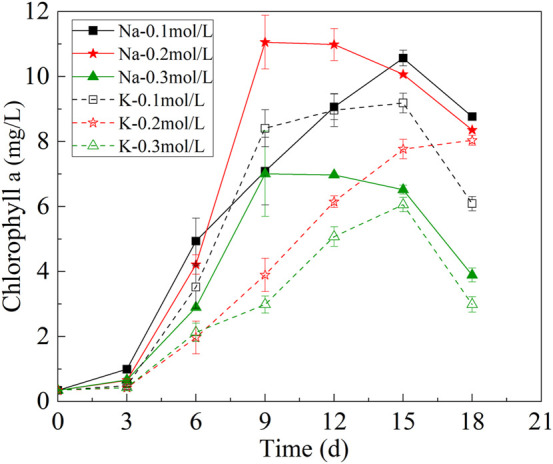
Changes of chlorophyll a under different bicarbonate concentrations.

Chlorophyll a can be used as an indicator to evaluate the photosynthetic capacity of microalgae. The change of chlorophyll a content of mutagenic *Spirulina* was shown in Figure 3. At the first 9 days of culture, the chlorophyll a content of mutagenic *Spirulina* increased with the growth of biomass. In the Na-.2 mol/L group, the chlorophyll a content of mutagenic *Spirulina* reached the maximum on the 9^th^ day, reaching 11.05 mg/L. After 9 days of culture, the content of chlorophyll a of mutagenic *Spirulina* showed a downward trend. This may be related to the change of solution pH, which affects the activity of pigment synthetase and the synthesis of chlorophyll a. After 15 days of culture, the chlorophyll-a content of mutagenic *Spirulina* decreased obviously, which may be caused by the insufficient nitrogen source in the medium. Under the condition of nitrogen limitation, the activities of enzymes involved in the degradation of organic nitrogen sources in mutagenic *Spirulina* were enhanced, which could degrade the nitrogen pigments in the cells to maintain their normal metabolism (Pancha et al., 2014; Depraetere et al., 2015).

### 3.4 Changes of inorganic carbon content, utilization efficiency and fixation rate

**FIGURE 4:**
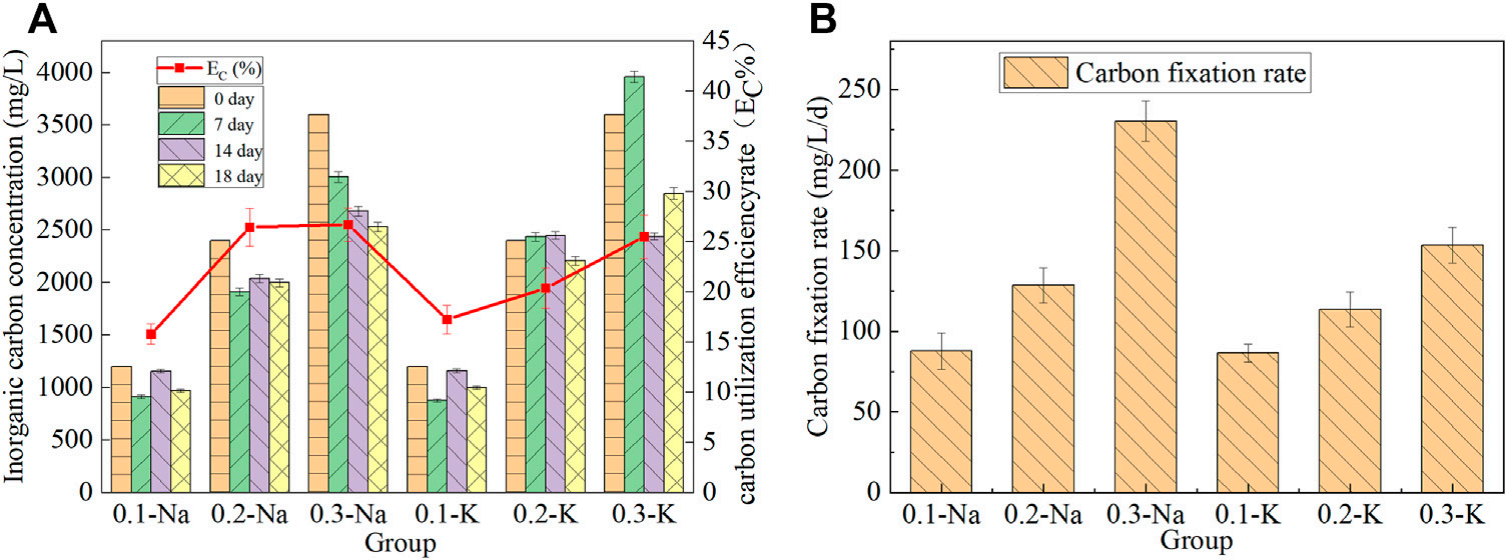
Chane of inorganic carbon content and utilization efficiency **(A)**, carbon fixation rate **(B)** under different bicarbonate concentration.

The concentration of inorganic carbon in the solution will change with the growth of mutagenic *Spirulina*, and the content of inorganic carbon and carbon sequestration rate in the solution of each group was shown in Figure 4A. After 18 days of culture, the inorganic carbon content of Na-.1 mol/L group decreased from 1,200 mg/L to 970 mg/L, and the carbon utilization efficiency was 15.79%. The concentration of inorganic carbon in Na-.2 mol/L group decreased from 2,400 mg/L to 1998 mg/L, and the carbon utilization efficiency was 26.45%. The highest carbon utilization efficiency (26.71%) was obtained in Na-.3 mol/L group, and the inorganic carbon concentration decreased from 3,600 mg/L to 2,531 mg/L. The carbon utilization efficiency of K-.1 mol/L, K-.2 mol/L and K-.3 mol/L groups were 17.25%, 20.38% and 25.49%, respectively. This indicated that NaHCO_3_ was more conducive to carbon utilization by mutagenic *Spirulina* in CAMC system.

The carbon fixation rate of mutagenic *Spirulina* in CAMC system was shown in Figure 4B. The carbon fixation rate of mutagenic *Spirulina* in Na-.1 mol/L, Na-.2 mol/L and Na-.3 mol/L groups were 87.83 mg/L/d, 128.55 mg/L/d and 230.36 mg/L/d, respectively. It was much higher than the 15 mg/L/d and 17 mg/L/d obtained by Song et al. who using NH_4_HCO_3_ as carbon source (Song C. et al., 2019). (Khoobkar et al., 2022) reported similar carbon fixation rate when cultivating *Chlorella* sp. in a photobioreactor. While the carbon fixation rate of mutagenic *Spirulina* in K-.1 mol/L, K-.2 mol/L and K-.3 mol/L groups were 86.58 mg/L/d, 113.74 mg/L/d and 153.41 mg/L. This indicated that the carbon fixation rate in NaHCO_3_ system was higher than that in KHCO_3_ system, which was also consistent with the dry weight of biomass.

### 3.5 Value-added components yield of CO_2_ absorption hybrid with microalgae conversion system

#### 3.5.1 Change of lipid content and yield

**FIGURE 5:**
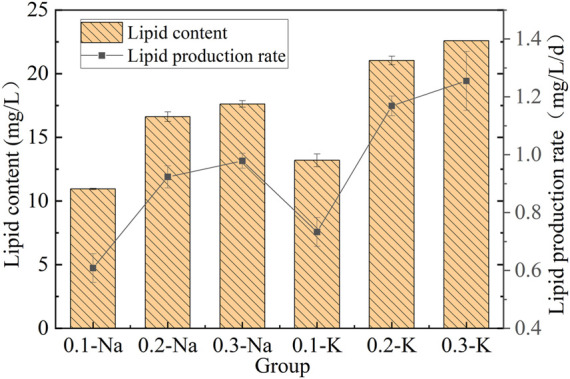
Lipid content and yield under different bicarbonate concentrations.

The lipid content and lipid production yield of mutagenic *Spirulina* in the CAMC system with different inorganic carbon concentrations was shown in Figure 5. After 18 days of culture, the contents of lipid in Na-.1 mol/L, Na-.2 mol/L and Na-.3 mol/L groups were 10.95 mg/L, 16.62 mg/L and 17.62 mg/L, respectively. While that of K-.1 mol/L, K-.2 mol/L and K-.3 mol/L groups were 13.20 mg/L, 21.04 mg/L and 22.60 mg/L. In the NaHCO_3_ system, the mutated *Spirulina* obtained the highest lipid production rate (.98 mg/L/d) at .3 Na-mol/L group. In K-.1 mol/L, K-.2 mol/L and K-.3 mol/L groups, the lipid production rate of mutated *Spirulina* was .73, 1.17 and 1.26 mg/L/d, respectively. High concentrations of Na^+^ and K^+^ contribute to lipid accumulation, which may be related to the stress effect. Previous study showed that salinity stress has positive impact on lipid synthesis (Mohan & Devi, 2014). The results showed that KHCO_3_ was beneficial for lipid synthesis in CAMC system, compared with NaHCO_3_.

#### 3.5.2 Change of polysaccharide content and yield

**FIGURE 6:**
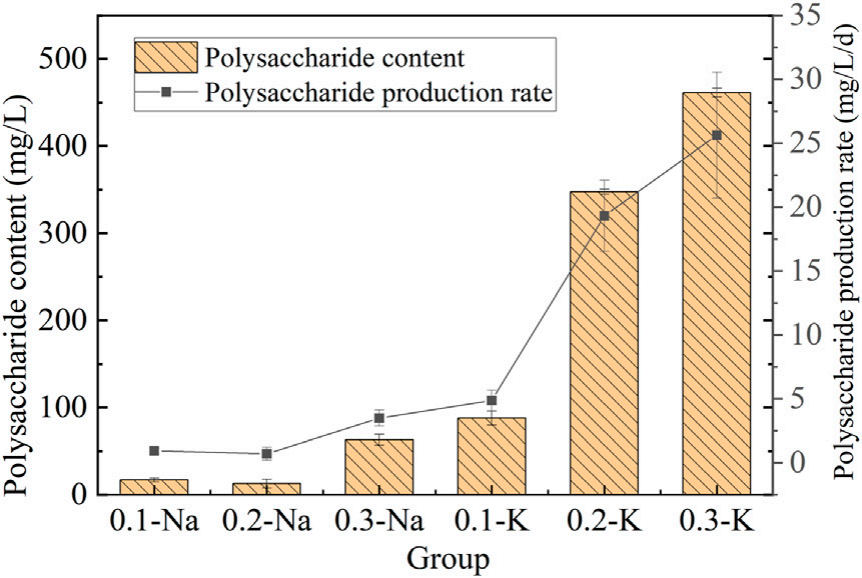
Polysaccharide content and yield under different bicarbonate concentrations.

Polysaccharide is a functional macromolecule with antioxidant, antiviral, immunomodulatory and anti-inflammatory activities (Sathasivam et al., 2019). The polysaccharide content of mutagenic *Spirulina* in CAMC system was shown in Figure 6. The polysaccharide content in the Na-.1 mol/L and Na-.2 mol/L group were 17.01 mg/L and 12.78 mg/L, respectively, and the highest polysaccharide content was obtained in the Na-.3 mol/L group (62.97 mg/L). The polysaccharide content of mutagenic *Spirulina* in K-.1 mol/L, K-.2 mol/L and K-.3 mol/L groups were 87.80 mg/L, 347.70 mg/L and 461.28 mg/L. Among all groups, the polysaccharide productivity of mutagenic *Spirulina* in K-.3 mol/L group was the highest, which was 25.63 mg/L/d. The results showed that compared with NaHCO_3_, KHCO_3_ was more conducive to polysaccharide synthesis, and .3 mol/L promoted the most.

#### 3.5.3 Change of protein content

**FIGURE 7:**
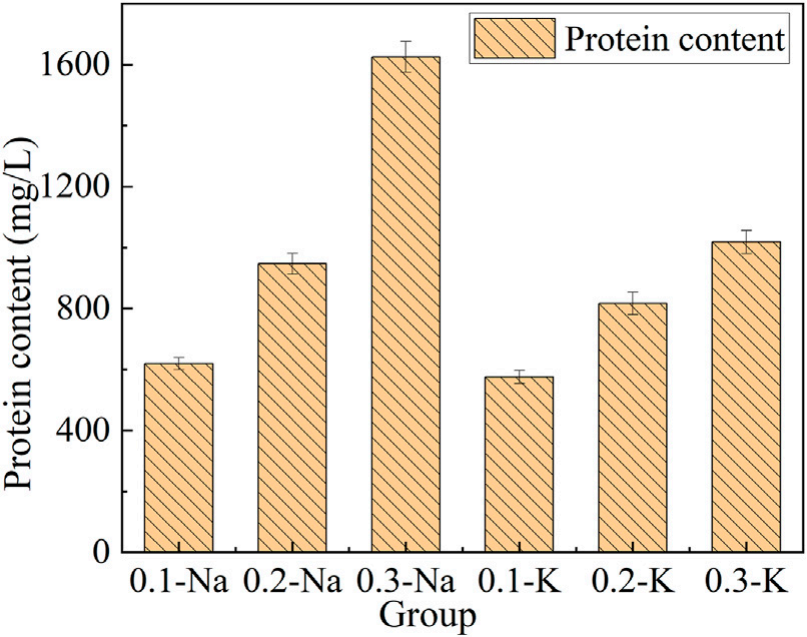
Protein content and yield under different bicarbonate concentrations.

Figure 7. Showed the protein content of mutagenic *Spirulina* in CAMC system. Under the same HCO_3_^−^ concentration, the protein content in Na^+^ system was higher than that in K^+^ system, which may be because Na^+^ can transmembrane into cells under the action of transporters (Reinfelder, 2011). The protein content of mutagenic *Spirulina* in Na-.3 mol/L group was the highest (1,625.68 mg/L). This maybe more transporters were needed to complete the transport of Na^+^ in a high concentration Na^+^ system, and microalgae will preferentially use more energy to synthesize this transporter, leading to an increase in protein content (Tsuji et al., 2021). This also further revealed the reason why the content of polysaccharide in NaHCO_3_ system was not as high as that in KHCO_3_ system (Figure 6). More energy was used for protein synthesis, resulting in less energy for polysaccharide synthesis (Pereira et al., 2019).

## 4 Conclusion

In this study, the chemical absorbent NaHCO_3_ and KHCO_3_ (represent products after Na_2_CO_3_ and K_2_CO_3_ fully absorb CO_2_) was selected as the research object, and the corresponding bicarbonate was used for the cultivation of mutagenic *Spirulina* to achieve carbon capture and resource utilization. Compared with KHCO_3_, NaHCO_3_ was conductive to the growth of mutagenic *Spirulina*, and .3 mol/L NaHCO_3_ promoted the biomass accumulation and carbon sequestration efficiency of CAMC system (reaching 2.24 g/L and 26.71%). KHCO_3_ promoted the production of lipid and polysaccharide, and NaHCO_3_ was conducive to the accumulation of protein (reaching 1,625.68 mg/L). In the subsequent application process, Na_2_CO_3_ or K_2_CO_3_ can be selected as the absorbent of CAMC system to cultivate mutagenic *Spirulina* according to the requirements of the target products. It is worth noting that a variety of new CO_2_ absorbents have been developed in recent years, and their feasibility and operating effects in coupled systems need to be continuously paid attention to.

## Data Availability

The original contributions presented in the study are included in the article/Supplementary Material, further inquiries can be directed to the corresponding author.
